# Association between Blood Pressure, Blood Pressure Variability, and Post-Stroke Cognitive Impairment

**DOI:** 10.3390/biomedicines9070773

**Published:** 2021-07-02

**Authors:** Kang-Po Lee, Alice Y. W. Chang, Pi-Shan Sung

**Affiliations:** 1 Department of Neurology, National Cheng Kung University Hospital, College of Medicine, National Cheng Kung University, Tainan 704, Taiwan; gavin.righ@gmail.com; 2 Department of Neurology, E-DA Hospital, Kaohsiung 824, Taiwan; 3 Department of Physiology, College of Medicine, National Cheng Kung University, Tainan 704, Taiwan; aywchang@mail.ncku.edu.tw; 4 Institute of Basic Medical Sciences, College of Medicine, National Cheng Kung University, Tainan 704, Taiwan; 5 Institute of Clinical Medicine, College of Medicine, National Cheng Kung University, Tainan 704, Taiwan

**Keywords:** post-stroke cognitive impairment, dementia, stroke, pathophysiology, treatments, blood pressure, blood pressure variability

## Abstract

After stroke, dynamic changes take place from necrotic-apoptotic continuum, inflammatory response to poststroke neurogenesis, and remodeling of the network. These changes and baseline brain pathology such as small vessel disease (SVD) and amyloid burden may be associated with the occurrence of early or late poststroke cognitive impairment (PSCI) or dementia (PSD), which affect not only stroke victims but also their families and even society. We reviewed the current concepts and understanding of the pathophysiology for PSCI/PSD and identified useful tools for the diagnosis and the prediction of PSCI in serological, CSF, and image characteristics. Then, we untangled their relationships with blood pressure (BP) and blood pressure variability (BPV), important but often overlooked risk factors for PSCI/PSD. Finally, we provided evidence for the modifying effects of BP and BPV on PSCI as well as pharmacological and non-pharmacological interventions and life style modification for PSCI/PSD prevention and treatment.

## 1. Introduction

Stroke ranks second worldwide for mortality and morbidity, and ischemic stroke accounts for 67.1–87% of all strokes [1,2,3]. Stroke causes a very large economic burden, approximately EUR 20–27 billion annually in the European region [4], with costs in the U.S. as high as USD 4000 per patient per month [5]. Moreover, in addition to the focal deficit-induced disability, there is the later cognitive impairment and behavioral-emotional dysfunction after stroke, which are easily overlooked. Previous studies suggested 9.2–31.4% of patients may have poststroke dementia (PSD). Poststroke cognitive impairment (PSCI) may be even higher at 35–47% [6,7,8,9,10] or as high as 80%, as was seen among a series of Chinese patients [11]. Poststroke patients with cognitive impairment have an independent risk of higher mortality [12]. The most vulnerable cognitive domains affected by stroke are working memory and episodic memory, motor dexterity and verbal fluency, followed by recognition memory [9,13]. Regarding the behavioral-emotional dysfunction after stroke, one-third of stroke patients suffer from poststroke depression as well as other neuropsychiatric domains, such as apathy, depression, and sleep disorder [14].

Poststroke cognitive function is not constant but dynamic. Stroke survivors may experience a sharp decline in cognitive function and neuropsychiatric symptoms immediately after a stroke. Early PSCI may become evident as early as 2 weeks afterwards [15]. Some patients may recover and return to their baseline condition between 3 and 6 months, but others do not recover [16]. Except for early PSCI, some individuals may again have an accelerated rate of decline, causing late PSCI [17,18,19,20,21]. Regarding which domains are more vulnerable, the results are controversial. One study suggested that, in early PSCI, the impairment is specifically related to the lesion site, whereas late PSCI is more of a global dysfunction; on the other hand, other studies suggested that, with the exception of perceptual motor function, all cognitive domains are impaired in early PSCI, especially memory. In late PSCI, executive and language function as well as visuospatial function may improve from 3 months to 1.5 years after the index stroke [15,22,23], while working memory may eventually recover years after the previous stroke [23].

In this review, we focused on the cerebral changes after a stroke and its relationship with the onset of PSCI. We provided the potentially useful markers to predict the occurrence of PSCI. Then, we untangled the relationships between blood pressure/blood pressure variability (BP/BPV) and PSCI and discussed the potential modifications and interventions.

## 2. Method and Data Source

A Medline (PubMed) literature review was performed by using the following search queries:

- (Post-stroke cognitive impairment) AND (Blood pressure)
- (Poststroke cognitive impairment) AND (Blood pressure)
- (Post-stroke cognitive impairment) AND (Blood pressure variability)
- (Poststroke cognitive impairment) AND (Blood pressure variability)
- (Post-stroke dementia) AND (Blood pressure)
- (Poststroke dementia) AND (Blood pressure)
- (Post-stroke dementia) AND (Blood pressure variability)
- (Poststroke dementia) AND (Blood pressure variability)
- (Cognitive impairment after stroke) AND (Blood pressure variability)
- (Dementia) AND (Blood pressure variability)

Two independent authors (K.-P.L. and P.-S.S.) screened titles and abstracts of the publications. Disagreements were solved by the consensus meeting among the three authors. Duplicated entries, retracted publications, studies on other diseases or focusing on special groups, works on animals or in vitro, studies without statistical analysis, non-English written papers, publications as commentaries, letters and editorials, and any other article that did not fit within the scope of this review were excluded. Articles listed in the references were also reviewed in search of more data. We only considered studies published within the last decade. 

**Figure 1:**
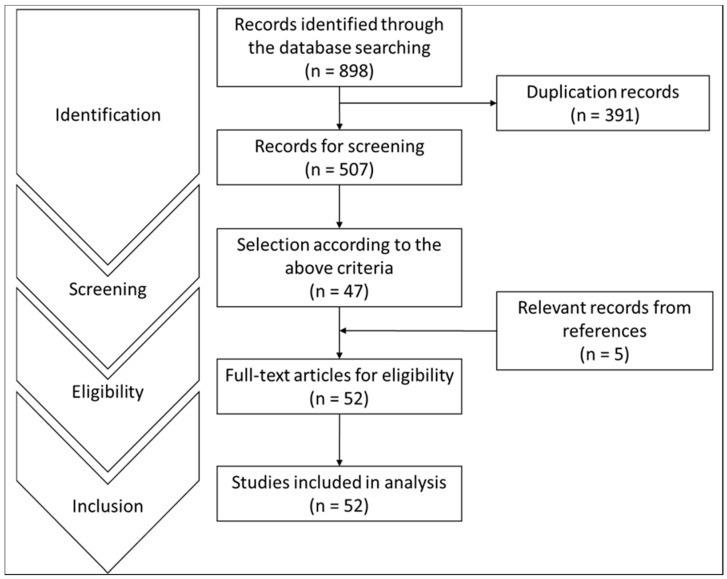
Flow diagram showing the search strategy.

A total of 898 results were retrieved. Among them, 47 publications were selected according to the aforementioned inclusion and exclusion criteria. The examination of the references from relevant papers detected five other studies fitting the purpose of this review. Therefore, a total of 52 papers were eventually included (Figure 1).

## 3. Anatomical, Biochemical and Pathological Changes after Stroke and Their Relationship with PSCI/PSD

Regarding PSCI/PSD right after stroke, it should first be noted that the location and the size of the infarction matters. A larger infarction size and some specific lesions (e.g., those involving angular gyrus, hippocampus, and frontal cortex) can cause immediate cognitive impairment and even dementia immediately after stroke [24]. A 2009 paper on pathology [25] reported that the neurons became eosinophilic in cytoplasm with a basophilic nuclei right after stroke. The neuron then degraded into debris, and the debris was phagocytized by macrophages, ranging in 1 day to 2 months. Not only neurons but also astrocytes experienced morphological changes. At first, astrocyte swelling was noted, and astrocytes carrying a necrotic aspect became gemistocytic around 2 weeks. Finally, an astrocyte alone with the neurons was necrotized and liquefied. Except for necrotic change, newer studies demonstrated that injured cells underwent apoptosis. They suggested that the so-called apoptosis–necrosis continuum would be a better nomination [26]. Moreover, the vessels also changed within a day, leading to plasma extravasation cellular infiltration, disintegration, and perivascular edema.

The earliest inflammatory response occurred about 4–6 h after onset, starting with leukocyte infiltration and followed by monocytes around 72 h later. Then, between 1 and 2 weeks, macrophages became prominent. The inflammatory response involved abundant cytokines, both pro-inflammatory (interferon (IFN)-γ, interleukin (IL)-6, IL-1β, tumor necrosis factor (TNF)- α and IL-17) and anti-inflammatory cytokines such as IL-10 [27]. These inflammatory responses may harm the injured brain, causing early cognitive impairment [28,29]. A possible explanation is that hippocampal pyramidal cells are selectively more vulnerable than other structures to inflammation. A damaged hippocampus and entorhinal cortex subsequently leads to cognitive impairment [30,31,32]. The inflammation may not be transient because a neuroimage study demonstrated inflammatory responses beyond 6 months after a stroke [33].

Then, the neurogenesis begins. Neurogenesis after stroke occurs in the subventricular zone (SVZ) and the hippocampal subgranular zone (SGZ) [34]. The SVZ is crucial for functional repairment, and the SGZ is crucial for cognitive function [35,36]. The neurogenesis may be both ipsilateral and contralateral to the lesion site. Despite trying to restore their function, aberrant neurogenesis causes further damages. The integration of these new and established cells may remodel the hippocampal network, leading to destabilization and even the loss of pre-stored memory [34,37]. The neurogenesis in SGZ started about 1 week after stroke, peaking around 2 weeks, then gradually recovered to baseline at around 1 month with dendritic maturation [35,38], but the aberrant neurons may have long-lasting effects after stroke [34]. In addition, animal models showed that inhibition of aberrant poststroke hippocampal neurogenesis reduced the remote memory deficits after stroke [38]. Although controversial results exist regarding the issue of poststroke hippocampal neurogenesis and poststroke cognitive outcomes in animal models due to different experimental design and behavior paradigms, poststroke hippocampal damage may be associated with the onset of PSCI/PSD. In humans, accelerated hippocampal atrophy following a stroke was noted both ipsilaterally and contralaterally to the lesion site at 1 year follow up [39,40]. Furthermore, more remote events may impair the hippocampal integrity [41]. 

In addition to the late stroke effect on the hippocampus itself, stroke may disrupt other structures as well. Lesions may involve so-called rich clubs, interrupting the hemispheric connections and the fronto-basal circuits, and may even cause cortical thinning and a decline in total brain volume [42,43,44,45]. A functional magnetic resonance imaging (MRI) study in stroke patients showed decreased functional connectivity and regional coherence due to lesions that interrupted white matter circuits, especially in the bilateral mesial temporal lobe and the posterior cingulate gyrus [46,47,48]. For unknown reasons, white matter integrity on the contralateral side was also affected, which had a more global negative effect on cognition [49]. 

The development of PSCI/PSD after stroke, therefore, depends on several factors: abovementioned stroke location and stroke volume, related neuronal damage, duration of neuroinflammation, and potentially aberrant hippocampal neurogenesis. In addition, the presence of other pre-existing or subsequent cerebral pathologies is also relevant [50]. 

The impact of Alzheimer’s disease (AD) pathology and changes associated with small vessel disease (SVD) on early and late PSCI is widely discussed. SVD changes and AD pathology are pathological brain changes that may pre-exist or later develop in the brain following a stroke, especially in the elderly. At stroke onset, these pathological changes were possibly subclinical or associated with various severities of prestroke cognitive impairment. In one review, it was suggested that these changes may represent reduced brain resilience to stroke and vascular pathology [17]. However, whether SVD burden, hippocampal atrophy, or amyloid burden correlate the most with PSCI/PSD remains controversial and conflicting [10,51,52,53,54,55,56,57,58,59,60,61]. One review concluded that AD pathology is an important contributor to prestroke dementia. In addition, the presence of AD-like retention shown on amyloid positron emission tomography (PET) was noted in approximately 30% of patients with early PSCI [17,62], which was significantly higher than that in age-matched nondemented patients following stroke or transient ischemic attack (TIA). Thus, AD pathology may be associated more with early PSCI/PSD. Regarding the impact of SVD, the presence of cerebral microbleeds (CMBs) and white matter hyperintensity (WMHs) were also both shown to be associated with early onset PSCI [17,32,63]. However, regarding late PSCI, evidence may suggest that the presence of severe SVD changes, such as three or more lacunes or severe WMH, was a strong predictor for late PSCI/PSD [64,65,66]. Another study showed that hippocampal AD pathology did not differ between demented or nondemented poststroke patients and suggested that a non-Alzheimer neurodegenerative process may play a role in dementia following stroke (the mean time from stroke to brain donor was 59–60 months) [55]. AD-like retention of the PET radiotracer in late PSCI was also lower than the retention in patients with early PSCI (19% over 3 year follow-up vs. approximately 30% over 3–6 months) [62,67]. However, those with amyloid burden seemed to have a more severe and rapid cognitive decline than those without amyloid pathology [67]. The contribution of AD pathology and SVD changes to the appearance of early and late PSCI warrants more investigation. 

## 4. Biomarkers for PSCI/PSD

### 4.1. Serological Biomarkers

Unlike animal models, human brain pathologies are precious and scarce. However, due to medical advances, more and more biomarkers are now available not only for vascular dementia but also possibly for predicting the risk of PSCI/PSD. S100B (calcium binding protein B), the S100B/asymmetric dimethylarginine ratio, and the homocysteine level correlated with baseline small vessel disease burden as leukoaraiosis, lacunar infarct, and deep microbleeds. These findings also lead to a higher risk of vascular dementia (VaD) [68,69]. A study demonstrated that, after a strategic stroke, there may be a surge in serum citrulline and dimethylarginine (DMA), and these surges, together with arginine depletion, independently correlate with low MMSE and predict the possibility of cognitive impairment [70]. Higher serum metabolites of glutamine, kynurenine, and lysophosphatidylcholine (18:2), uric acid (UA), low density lipoprotein-cholesterol (LDL-C), vascular endothelial growth factor (VEGF), cortisol, plasma fibrinogen, as well as low plasma amyloid Aβ42, low folic acid, and low vitamin B12 may predict PSCI [18,71,72,73,74,75,76,77,78,79,80,81]. A panel of glutamine, kynurenine, and lysophosphatidylcholines was proposed for predicting PSCI [73]; however, cholesterol level as a predictor is controversial [80,82]. Elevated urine formaldehyde, indicating that higher oxidative stress causes PSCI, was reported in one study [83]. Inflammatory markers are also important. Cerebrospinal fluid (CSF) IL-6, -8, -10, and -1β as well as more conventional markers such as C-reactive protein (CRP), highly sensitive CRP (hs-CRP), and rheumatic factors are associated with PSCI [84]. Elevated CRP and hs-CRP during the acute phase may even predict PSCI up to 1 year after stroke [20,76]. The APOE ε4 allele was thought to be an independent risk factor for PSCI and BDNF Val<Met polymorphism as a protector [85,86,87,88], but the Bahraini cohort suggested no such correlation between APOE ε4 and PSCI [89]. 

### 4.2. Imaging Biomarkers

Almost all stroke patients underwent brain imaging, which told us not only the exact lesion sites but also the baseline brain reserve. Low grey matter, especially over the frontal cortex, WMH burden and location, and brain atrophy strongly predicted future PSCI [26,60,90]. In a brain with a high WMH burden, subsequent small infarcts may interrupt vulnerable projections and lead to the thinning of cortical grey matter, thus leading to PSCI [45,91]. Regarding PSCI and SVD changes, early PSCI is related to cerebral microbleeds (CMBs) and WMH [17,32,63], and severe WMH or three or more lacunes was associated with the onset of late PSCI [64,65,66]. A functional MRI using the default mode showed decreased functional connectivity in left medial temporal lobe, posterior cingulate, medial prefrontal cortical areas, and bilateral hippocampus in PSCI patients [47,48,92].

As mentioned above, those with amyloid burden on amyloid PET seemed to have a more severe and rapid cognitive decline after a stroke than those without amyloid pathology [67]. However, another study suggested the occurrence of late PSCI has less connection to amyloid pathology [93]. An ongoing trial, the Determinants of Dementia After Stroke (DEDEMAS) study is still ongoing and may tell us more about the relationship between amyloid burden on amyloid PET and PSCI [94]. Tau PET, on the contrary, may indicate reactive gliosis essential for functional repair. A study suggested that, in amyloid-negative stroke patients, a high uptake of tau was noted both in the lesion and the perilesional area, and the level correlated with the patient’s cognitive performance [95]. 

## 5. The Relationship between BP, BPV, and PSCI/PSD 

For a stroke patient, BP control is important, but more and more studies focused on BPV over the last decade. Previous studies showed that higher visit-to-visit BPV predicted subsequent stroke in TIA patients [96], higher stroke risk [97], and higher mortality after stroke [98,99]. However, the relationship between BP and BPV and cognitive function is less discussed. Here, we pointed out current knowledge on this tangled relationship among BP, BPV, and cognitive impairment. 

### 5.1. The Association between BP and Cognition

Regarding BP and dementia risk, population-based studies showed that, from midlife to the geriatric stage, hypertension is correlated with future dementia, including both AD and VaD [100,101,102,103,104,105,106,107,108,109,110]. Another large review comprising 17 different systemic reviews concluded that hypertension leads to a higher risk of VaD and cognitive decline but to less AD [111]. However, other studies suggested that no relation between hypertension and dementia exists [112,113] or that the association is the reverse [114]. Additionally, the relation between cognition and BP may vary by age [115,116]. 

Higher systolic blood pressure (SBP) may have a negative effect on the hippocampus and the dentate gyrus [117,118,119], thus negatively influencing the brain reserve. The effect of SBP may also start in early adulthood and predict a lower hippocampal volume in the geriatric stage [119,120]. Hypertension might interrupt the connectivity of the temporal lobe, the thalamus, the prefrontal cortex, and especially the hippocampus [117]. Not only hippocampal volume but also hypertension are widely believed to be risk factors for the changes associated with SVD [121,122,123]. Elevated diastolic BP may be a more specific indicator of the presence of WMHs [124]. In addition to causing SVD changes, in a rat model, hypertension increased amyloid deposition not only in the brain parenchyma but also in the vessel walls in the fronto-parietal region and the hippocampus [125,126]. In APOE ε4 non-demented individuals, elevated blood pressure was also associated with decreased hippocampal volume and a deterioration in cognitive function processing speed [127].

Although there were many observational studies, the true mechanisms underlying the changes are not completely understood. For example, how does hypertension negatively affect our brain despite cerebral autoregulation? It may be that the arterial remodeling in a chronic hypertensive situation causes vessel wall stiffness, which impairs autoregulation. Moreover, in addition to large arteries, hypertension may also damage arterioles and capillaries [128,129,130]. The above changes may lead to decreased cerebral blood flow (CBF) and cerebral hypoperfusion [131]. In animal models, increased arterial stiffness induced brain dysfunction [130]. A newer concept of the neurovascular unit (NVU) emphasized on the bidirectional talk of neurons and vessels for cerebral autoregulation [132]. A mouse study clearly demonstrated that hypertension may lead to NVU dysregulation and cerebrovascular injury [133,134]. Elevated BP may also increase the inflammatory process and breakdown of the blood–brain barrier (BBB) [135,136,137,138], which may impair Aβ clearance via the NVU, leading to AD [139].

In addition to the relationship between hypertension and cognitive dysfunction, hypotension would be another potential risk. In patients with down syndrome, lower baseline BP compared with general population was noted [140]. Even with the lack of hypertension or prehypertension, they still have much higher rates of cerebrovascular disease and dementia [141,142]. Another large-scale community-based cohort study with long-term follow-up showed that midlife hypertension and late-life hypotension were associated with 1.62-fold risk of later onset dementia compared with those who were normotensive in mid- to late-life. In addition, declines in BP preceded the onset of mild cognitive impairment or dementia [143]. Whether hypotension plays a role in the etiology of neurodegenerative disease or is a potential concomitant phenomenon of neurodegenerative process warrants future research.

### 5.2. The Association between BPV and Cognition

BPV is not a constant value but a dynamic value that changes over time, and these changes are classified as ultrashort-term BPV, short-term BPV, and long-term BPV, with each correlating, respectively, to beat-to-beat variations, variation within a day and day-to-day, and visit-to-visit variation [144]. The principal indices of these measures include standard deviations, coefficient variations, ranges, night/day ratios, and many others [144,145]. BPV may be another important factor related to cognitive changes, but lack of consensus on BPV methods resulted in heterogeneity among previous epidemiological BPV studies and cognition, and increasing evidence suggests that BPV may be more important than hypertension itself. 

In the dementia-free middle-age/old-age population, higher systolic BPV (SBPV) is associated with a higher risk of dementia, and the relationship increases with the duration of higher SBPV [146,147,148]. In a community-dwelling old population, increased short-term BPV, SBPV, diastolic BPV (DBPV), and day-to-day BPV but not higher definite BP were associated with cognitive decline and dementia [149,150,151,152,153,154,155,156,157]. However, controversial results were found in other studies [158,159]. As in BP and cognition, age may also play a role. Increased long-term BPV leads to a higher risk of cognitive dysfunction only in the middle-aged population but not in geriatric patients [160]; however, some studies mentioned such correlation in long-term BPV and cognitive impairment in the geriatric population [153,161,162]. Both diastolic BPV (DBPV) and SBPV may be associated with more severe deterioration in cognitive function, and there may be a synergistic effect if both are increased [163,164,165,166]. In patients with AD, increased short-term BPV and long-term BPV were associated with faster deterioration [150,167]. Higher long-term BPV is associated with AD and amnestic mild cognitive impairment (aMCI) [168]. In patients diagnosed with mild to moderate AD, both higher SBPV and DBPV lead to faster deterioration of cognitive function [169], but in patients with frontotemporal dementia (FTD), no such correlation was noted. A 2018 review suggested that higher long-term BPV is associated with poorer cognitive function in both the normal population and demented patients [170]. 

However, the true mechanisms underlying the relationships between BPV and the decline in cognitive function remain uncertain [171]. BPV is thought to induce systemic microvascular dysfunction, but the effect on the cerebral vasculature is less certain [172]. In patients with atrial fibrillation, numerous episodes of repetitive hypoperfusion and hyperperfusion were noted in arteries and even in the cerebral capillary system when deep white matter underwent ischemic change [173,174,175]. The above mechanism may also be considered to apply to high BPV patients as well. Moreover, an animal model showed that increased BPV without hypertension caused vessel wall thickness and subsequent left ventricular hypertrophy [176]. The overall pathophysiology was possibly due to hemodynamic instability induced perfusion imbalance and subsequent inflammatory processes and endothelial damage. These changes were shown to lead to the thickening of blood vessels, arterial stiffness, and deposition of Aβ [170,177]. In addition to Aβ deposition, a higher visit-to-visit SBPV may lead to decreased white matter microstructural integrity and an increased rate of brain atrophy [178]. More specifically, long-term BPV may have a remote negative effect on hippocampal volume, especially if it begins in young adulthood [179]. In patients with and without cognitive function complaints, increased daily BPV and day-to-day SBPV were associated with a higher burden of SVD changes, but the correlation was less significant with DBPV [149,180,181]. One study found that increased BPV was associated with total WMH but not with either periventricular or deep WMHs [182]. However, the same group subsequently conducted a meta-analysis showing that BPV was independently associated with a higher SVD burden, especially total WMHs and periventricular WMH but not deep WMH [183]. Another study group demonstrated that a high SBPV was associated with WMH, lacunes, and microbleeds [184]. A meta-analysis of people without dementia showed that BPV, regardless of the time span, was independently correlated with WMH, but the relationship was less evident with lacunes and microbleeds [185]. The association between BPV and SVD changes varied among studies, which was potentially related to different study designs and targeted populations. Table 1 summarized the relationship between BP/BPV and cognitive function. 

### 5.3. Influence of BP/BPV in PSCI and PSD

Regarding BP/BPV and PSCI, the situation is more complicated but less frequently discussed. A higher SBP upon stroke admission seemed to be correlated with a higher risk of PSCI [186]. In the acute phase within 7 days, there seemed to be a U-shaped association regarding SBP and poststroke cognition [187]. In the subacute phase of less than 90 days, elevated SBP was thought to be a risk factor for PSCI with or without DBP differences, and DBP was significantly inversely related to cognition after adjusting for age, education, and races [188]. In the chronic phase, home BP is more important than clinic BP, and higher morning BP and bedtime BP were specifically related to a higher risk for recurrent ischemic events and PSCI [189]. However, the Sydney study suggests that hypertension is not a risk factor for PSCI between 3 and 6 months [190]. A study on BP control also showed no beneficial effect on cognition for different levels of SBP [191], and another demonstrated that the correlation between hypertension and PSCI was established only when patients had concurrent hyperhomocysteinemia [192]. In fact, a meta-analysis stated that BP and poststroke cognitive performance had no correlation [7]. However, increased BPV during the early phase of stroke may predict PSCI [193,194]. The subgroup analysis of the “PreventIon of CArdiovascular events in iSchemic Stroke patients with high risk of cerebral hemOrrhage (PICASSO)” trial showed that BPV, not SBP, was associated with faster cognitive decline after a stroke [195]. 

As mentioned above, BP or BPV may have a potential negative effect on poststroke cognitive function. BP and BPV are also potentially related to the major pathophysiology of PSCI, including AD pathology, SVD changes, and ischemic neurodegeneration. Further study may be warranted to investigate the impact of BP/BPV on the progression of PSCI/PSD. Table 2 summarized the relationship of BP/BPV and cognitive function after stroke. 

## 6. Management to Modify or Prevent PSCI

### 6.1. General Picture of BP Control in Stroke 

For the acute phase after a stroke, the patient usually experiences a period of elevated blood pressure due not only to autoregulation, which enhances cerebral perfusion to minimize the ischemic core, but also to acute stress, intracranial hypertension, and other systemic disarrangements [196]. In the acute phase, high BP was thought to be related to a worse clinical outcome in the Fukuoka study when it was 154/89 mmHg [196], but a better outcome was found in another where BP was 150/70 mmHg [197]. A higher BP may interrupt the BBB, resulting in worsening brain edema, but a lower BP means a decrease in cerebral perfusion and an extension of the infarct size [197]. Regarding antihypertensive drug trials for acute stroke, early initiation of angiotensin converting enzyme inhibitors (ACEIs), angiotensin receptor blockers (ARBs), adrenoreceptor antagonist (both alpha and beta), and CCB in the acute phase showed nearly neutral or negative findings for functional outcome or survival [198]. Recent American Heart Association (AHA) guidelines suggest that aggressive BP control may not be needed during the first 72 h, unless comorbid systemic disorders warrant strict BP control, such as a hypertensive crisis causing organ damage, intravenous thrombolytic or endovascular therapy with successful recanalization, or a BP higher than 220/120 mmHg [199]. European Stroke Organization (ESO) guidelines suggested no aggressive control when BP is lower than 220/110 mmHg in the first 24 h of stroke onset [200].

For secondary prevention, AHA guidelines suggest keeping office BP below 130/80 mmHg using thiazide, ACEI, and ARB [201]. 

### 6.2. Evidence Regarding Modifying the Potential Effect of BP/BPV on PSCI

Increased BPV or higher SBP may potentially raise the risk of PSCI, but the evidence regarding BP/BPV control and PSCI prevention is inconclusive. In animal studies, angiotensin receptor blockers (ARBs) effectively prevented Aβ42 toxicity, reactive microgliosis, and apoptotic cell death, leading to a reduced infarct size and prevention of PSCI following acute ischemic events within 30 days [202,203]. However, the human situation is more complicated. The appropriate time for medication seems to lead to controversial results. The Scandinavian Candesartan Acute Stroke Trial (SCAST) and the Valsartan Efficacy oN modesT blood pressUre Reduction in acute ischemic stroke (VENTURE) trial suggested early administration of ARBs may lead to worse functional outcomes or the risk of neurological deterioration [204,205]. Entering the subacute phase, ARBs were reported to be effective for preventing PSCI in one study [206] but not another [207]. Strict BP control (≤125 mmHg vs. ≤ 140 mmHg vs. ≤160 mmHg) also yielded no definite effect on the prevention of PSCI in the 1 year post-stroke period [191]. The Perindopril pROtection aGainst REcurrent Stroke Study (PROGRESS) showed a positive effect in cognitive function maintenance in a poststroke patient with a combination of ACEIs and indapamide that reduced the overall risk of PSCI by one-third [208]. In addition to ordinary antihypertensive medications, one study reported that tadalafil improved cerebral perfusion in patients with small vessel occlusion (SVO) [209]. However, there was no evaluation of cognition.

### 6.3. Other Pharmacological and Nonpharmacological Approach to Modify or Prevent PSCI

Statins showed a promising result in secondary stroke prevention, but the Heart Protection Study (HPS) and the PROspective Study of Pravastatin in the Elderly at Risk (PROSPER) showed that they had no positive impact on cognitive function [210,211]. One report suggested that a combination of piracetam and cinnarizine may improve late poststroke cognition in patients compared to those without treatment [212]. Cilostazol, an antiplatelet agent working on the P2Y12 receptor, is known for its antioxidative stress activity, neuroprotection (particularly toward hippocampal cells), and prevention of amyloid oligomerization [213,214]. One Japanese study demonstrated that it may facilitate cognitive recovery in the subacute phase beyond 1 month [215], but a study by a Korean group suggested it had no better effect on poststroke cognition compared to aspirin [216]. 

Acetylcholinesterase inhibitors are effective AD medications, and their efficacy was also tested in PSCI. Two randomized controlled trials focusing on late PSCI demonstrated significant cognitive recovery with these medications compared with a placebo [217,218]. Finally, a preliminary study showed a promising effect of amantadine on neurocognitive function [219].

Other than medication, there are some other methods to modify or prevent PSCI. Acupuncture was reported to be effective in preventing PSCI in retrospective studies, randomized control trials, and meta-analyses [220,221,222]. Physical training was said to be effective in cognitive improvement in one review but inconclusive in another [223,224]. Early cognitive training is effective for memory and attention [224], but the long-term effect is uncertain [18]. 

Diet is also important. Alcohol consumption is controversial in dementia patients: a light to moderate amount has a protective effect, but an excessive amount leads to direct neurotoxicity [225]. Alcohol consumption is also thought to be a risk factor for PSCI [71], and in PSD patients, it carried a higher risk of recurrent stroke [226]. On the other hand, coffee drinking, especially mocha, showed protective effects against poststroke dementia in subcortical infarction patients, but smoking may eliminate them [227]. The nutritional supplements cichotoline and L-acetyl carnitine were reported to have positive effects on improving poststroke cognitive performance and preventing poststroke cognitive impairment [18,228,229,230]. 

**Figure 2:**
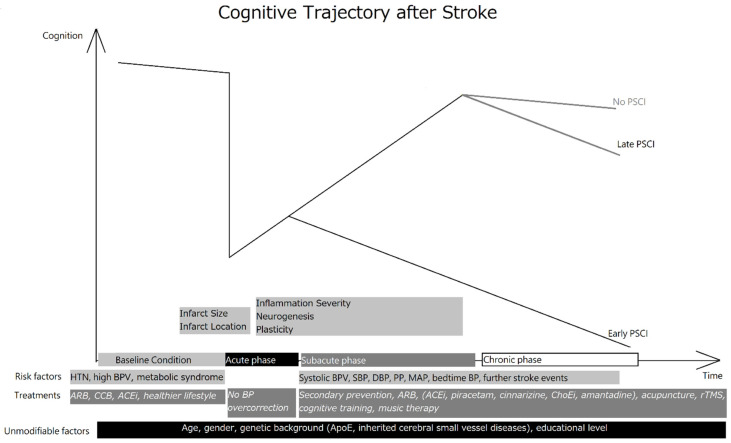
Cognitive trajectory after stroke.

In the PSCI group, repetitive transcranial magnetic stimulation (rTMS) seemed to improve patients’ activities of daily living (ADLs) and cognitive function by enhancing functional connectivity and the amplitude of low-frequency fluctuation in the bilateral mesial–prefrontal cortex, the cingulate gyrus, and many other brain regions [231,232]. Additionally, a systemic review found that excitatory or inhibitory stimulation of the dorsolateral prefrontal cortex (DLPFC) may improve patients’ attention, working memory, long-term memory, and cognitive function [233], but there was a marked heterogeneity in the enrolled studies. Nevertheless, an in vivo study showed that low frequency rTMS may upregulate hippocampal neuron synaptic plasticity via the BDNF-TrkB pathway [234,235]. In the hippocampus, Calb2, Zic1, Kcnk9, and Grin3a genes were upregulated as were the glutamatergic synapses [236]. Furthermore, rTMS may have a beneficial effect on poststroke depression as well [237]. Music therapy (MT) may have a positive effect, but the data are conflicting. Some studies suggested a positive effect on verbal memory, focused attention, and quality of life in poststroke patients with PSCI, but others showed no improvement in mood or cognition [238,239]. The Figure 2 summarized the poststroke cognitive changes, factors affecting the cognitive function, along with possible interventions and treatments.

## 7. Limitation

This review had several limitations. First, we enrolled mainly English-written studies, thus there was a certain degree of selection bias. Second, the studies and the reviews enrolled were highly heterogeneous. The definition of PSCI, its classification, diagnostic criteria, the parameters of BP, and the duration of follow up may have influenced the findings and the conclusions. Currently, a universally accepted consensus of this disease is still missing. Third, though we tried to figure out the underlying pathophysiology, there is a lack of human brain pathology or long-term functional imaging or pathology follow up to clarify the longitudinal change in a brain after a stroke.

## 8. Conclusions

Cognition alterations after a stroke are a complicated and dynamic process. Evidence suggests that a stroke may not only induce a cascade of inflammatory processes that interrupt circuits between cortical regions, leading to remote atrophic changes outside the lesion, but may also potentially interfere with the accumulation or the clearance of amyloids. The existence or the progression of AD pathology and SVD changes may contribute differently to early and late PSCI. The relationships between BP/BPV and PSCI/PSD are complicated and depend on particular parameters and different stages of stroke. BP or BPV may have a potential negative impact on the major pathophysiology of PSCI, including AD pathology, SVD changes, and ischemic neurodegeneration, and some evidence may suggest BP/BPV control for PSCI modification or prevention. Further studies may be warranted. A longitudinal study should be carried out that includes brain MRIs, functional imaging, and/or an amyloid or Tau PET to reveal the potential cerebral pathology interaction in PSCI patients. Large scale investigations into the definite effect of BP/BPV changes at different stages of a stroke and appearance and progression of PSCI/PSD with both short-term and long-term cognitive outcomes may be considered as well.

## Figures and Tables

**Table 1:** Summary of the relationship of blood pressure, blood pressure variability, and cognitive function.

Author and Year	Population	Measurement and Duration	Case Number	Outcome Measure	Blood Pressure Parameter	Result
Launer, L.J. et al. [100]	Middle-aged Japanese American, male	Prospective, 20–25 years follow up	3703 normal population	CASI, IQCODE	SBP, DBP	BP↑→Risk for dementia↑in drug naïve men
Kivipelto, M. et al. [101]	Mild-aged, Finnish	Prospective, 21 (SD 4.9) years follow up	1449 normal population	MMSE	SBP, DBP	SBP↑→Risk for dementia↑
Yamada M. et al. [102]	Geriatric, Japanese	Retrospective 25–30 years	No dementia: 1660 Dementia: 114	CASI, MMSE, Hasegawa Dementia Scale (HDS)	SBP	SBP↑→Risk for dementia↑
Whitmer, R.A et al. [103]	Middle-aged, American	Prospective 20–30 years follow up	8845 normal population	Diagnosis of Dementia	Diagnosis of hypertension	Hypertension →Risk for dementia↑
Yoshitake, T et al. [104]	Geriatric, Japanese	Prospective 7 years follow up	828 normal population	MMSE, HDS	SBP	SBP↑→Risk for dementia↑
Qin, H. et. al. [105]	Middle-aged to geriatric, Chinese	Prospective 7 years follow up	277 MCI patients	MMSE, MoCA, CDR	Diagnosis of hypertension	Hypertension →Risk for dementia↑
Zúñiga-Salazar, G.A., et al. [106]	Middle-aged, Ecuadorian	Cross section, observational	Hypertensive, non-demented 60	MoCA	SBP, DBP, Diagnosis of HTN	Hypertension duration↑→ MoCA score↓ SBP↑→ MoCA score↓
Bahchevanov, K.M., et al. [107]	Middle-aged, Bulgarian	Cross section	No dementia: 112	Consortium to Establish a Registry for Alzheimer’s disease Neuropsychological Battery (CERAD-NB)	Diagnosis of HTN	Hypertension → CERAD-NB score↓
Boo, Y.Y., et al. [108]	Middle-aged, Korean	Prospective 14 years follow up	4289 normal population	MMSE	BP	BP↑→Risk for dementia↑
Sun, D., et al. [109]	Middle-aged, American	Prospective 30 years follow up	1369 normal population	Verbal learning Test, Digital Symbol Substitution Test (DSST), Stroop Interference Test	SBP, DBP, PP	10 mmHg ↑in SBP, DBP, PP →DSST score↓
Shim, Y.S. and H.E. Shin [110]	Geriatric, Korean	Cross section	Impaired cognition: 174	MMSE	SBP, short-term BPV	SBP →↑→Risk for dementia↑
Kuller, L.H. et al. [112]	Geriatric, American	Retrospective	3608 normal population	MMSE, IQCODE	Diagnosis of hypertension	No relation between hypertension and dementia
Tyas, S.L. et al. [113]	Geriatric, Canadian	Prospective 7 years follow up	Normal cognition: 1335 Impaired cognition: 42	Modified MMSE	Diagnosis of hypertension	No relation between hypertension and dementia
Carmona-Abellan, M., et al. [114]	Middle-aged to geriatric, Spain	Retrospective >2.5 years follow up	Normal cognition: 2087	Diagnosis of dementia, MCI	SBP, DBP	↓SBP, BP → Risk of cognitive impairment ↑
Hestad, K., et al. [115]	Middle-aged to geriatric, Norwegian	Prospective 8 years follow up	4465 normal population	MMSE, Digit symbol Test, Twelve-word test	BP	Male ≤ 65↑SBP,↑DBP → Cognition↓; reverse in male >65 Female≤ 65↑SBP → Cognition ↑; reverse in female >65
Feng, R. et al. [117]	UK biobank	Cross section	Hypertensive: 2720 Normal BP: 12366	prospective memory, numeric memory, fluid intelligence, reaction time	Diagnosis of hypertension	Hypertension →Risk for dementia↑
Li, H. et al. [118]	Middle-aged, Chinese	Cross section	cognitive impairment: 59	MoCA, Stroop test, Verbal fluency test	SBP, SBP variability (SBPV)	↑SBP, SBP variability → ↓dentate gyrus volume
Walker, K.A., et al. [143]	Middle-aged, American	Prospective 24 years follow up	4761 normal population	Comprehensive neuropsychological battery, CDR, diagnosis of dementia	SBP, DBP	midlife hypertension and late-life hypotension → risk for dementia ↑
Ma, Y. et al. [146]	Geriatric, Dutch	Prospective 14 years follow up	Normal cognition: 5273	MMSE	Long-term SBPV, Long-term DBPV	↑long-term SBPV,↑DBPV→ Risk of cognitive impairment↑
Yano, Y. et al. [148]	Middle-aged, American	Prospective 25 years follow up	15792 normal population	Delay Word Recall Test, Digit Symbol Substitution Test, Word Fluency Test	SBP, DBP, SBPV, DBPV,	↑SBPV,↑DBPV→ Cognitive function ↓ SBP, DBP → No association
Godai Si, K. et al. [149]	Geriatric, Japanese	Cross section	111 normal population	MoCA	Short-term BPV	↑short-term BPV → Risk of cognitive impairment↑
de Haus, R.A.A. et al. [150]	Geriatric, Dutch	Prospective 1.5 years follow up	460 mild-to-moderate AD patients	Alzheimer’s Disease Assessment Scale–cognitive subscale (ADAS-cog)	Long-term BPV Short-term BPV	↑short-term BPV, ↑long-term BPV → Risk of cognitive impairment↑
Oishi, E. et al. [151]	Geriatric, Japanese	Prospective 5 years follow up	1674 normal population	MMSE, HDS	Day-to-day BPV Daily BPV SBP	↑Day-to-day BPV→ risk of cognitive impairment↑ ↑SBP →Risk of VaD↑
Cho, N. et al. [152]	Geriatric, Japanese	Cross section	232 normal population	MoCA	SBP, BPV	↑BPV → Risk of cognitive impairment↑
Fujiwara, T. et al. [153]	Geriatric, Japanese	Prospective 1 year follow up	524 normal population	Working memory test	Short-term BPV Long-term BPV	↑BPV → Risk of cognitive impairment↑
Liu, Z. et al. [154]	Geriatric, Chinese	Prospective 2.3 years follow up	248 normal population	MMSE	SBPV	↑SBPV → Speed of cognitive impairment↑
McDonald, C. et al. [155]	Geriatric, UK	Prospective 5 years follow up	353 normal population	MMSE, Cambridge Cognitive Examination (CAMCOG)	Day-time BPV	↑SBPV, DBPV → Risk of cognitive impairment↑, Speed of cognitive impairment↑
Nagai, M. et al. [156]	Geriatric, Japanese	Prospective 1 year follow up	201 patients high risk for CVD	MMSE	Long-term BPV Short-term BPV	↑long-term BPV → Risk of cognitive dysfunction↑
Yildirim, E. et al. [157]	Geriatric, Turkish	Prospective 1 year follow up	435 hypertensive patients	Standardized mini mental test (sMMT)	Short-term BPV	↑short-term BPV → Risk of cognitive dysfunction↑
Tsang, S. et al. [158]	Middle-aged to geriatric, American	Cross section	Normal cognition: 94	MMSE, Computer Assessment of Mild Cognitive Impairment (CAMCI)	SBPV, DBPV	No association of BPV and dementia
van Middelaar, T. et al. [159]	Geriatric, Dutch	Prospective 6.4 (SD 0.8) years follow up	2305 normal population	MMSE	Long-term BPV	No association of BPV and dementia
Qin, B. et al. [160]	Middle-aged to geriatric, Chinese	Prospective 3.2 years follow up	976 normal population	Telephone Interview for Cognitive Status–modified (TICS-m)	Long-term BPV	↑long-term BPV → Risk of cognitive dysfunction↑ in middle-aged; but no association in geriatric patients
Alpérovitch, A. et al. [161]	Geriatric, French	Prospective 8 years follow up	6506 normal population	MMSE	Long-term SBPV	↑SBPV → Risk of dementia↑
Epstein, N.U. et al. [162]	Middle-aged to geriatric, American	Prospective 3 years follow up	Normal cognition: 181 MCI 247	MMSE, CDR, ADAS-COG, Trail B, Digit Symbol Test, Rey auditory learning test	Long-term BPV	↑Long-term SBPV → Risk cognitive dysfunction ↑
Zhou, T.L. et al. [163]	Middle-aged to geriatric, American	Cross section	1804 normal population	Memory function Processing speed Executive function	Ultra-short BPV Daily BPV Long-term BPV	↑ultra-short, ↑daily SBPV, ↑DBPV → Cognitive performance↓
Rouch, L. et al. [164]	Geriatric, French	Prospective 3 years follow up	3319 normal population	MMSE	Long-term SBPV Long-term DBPV MAP, PPV	↑long-term SBPV, long-term DBPV → Risk of dementia↑
Sabayan, B. et al. [165]	Geriatric, European	Prospective 3.2 years follow up	5461 patients with CV risk without cognitive impairment	Stroop color and word test, letter-digit coding test, picture-word learning test,	SBP, DBP Long-term SBPV Long-term DBPV DBPV	↑long-term BPV → Risk of cognitive impairment↑
Yoo, J.E. et al. [166]	Korean data base	Retrospective 6.2 years follow up	7,844,814 patients	Diagnosis of dementia	SBPV, DBPV	↑long-term BPV → Risk of all dementia↑, AD↑, VaD↑
Lattanzi, S. et al. [167]	Geriatric, Italian	Prospective 12 months follow up	240 patients with dementia	MMSE	SBP, DBP, SBPV, DBPV	↑SBPV → Progression of cognitive decline↑
Sible, I.J. et al. [168]	Middle-aged to geriatric American and Canadian	Prospective 12 months follow up	681 normal cognition 479 MCI 261 AD	MMSE, CDR	SBP, DBP, BPV, Long-term BPV	In AD patients there is↑BPV ↑BPV → ↑progression in MCI
Lattanzi, S. et al. [169]	Geriatric, Italian	Prospective 12 months follow up	248 AD 81 FTD	MMSE	BP, BPV	↑SBPV → ↑progression in AD
Yano, Y. et al. [178]	Young adult, American	Prospective 25 years follow up	5115 normal population		SBP, DBP Long-term BPV	↑BPV in early age → ↓hippocampal volume and integrity

↑, increase; ↓. decrease; → leads to.

**Table 2:** Summary of the relationship among blood pressure, blood pressure variability, poststroke cognitive impairment, and poststroke dementia.

Author and Year	Follow up after Stroke	Case Numbers	Special Condition	Outcome Measure	Blood Pressure	Result
Gong, L. et al. [186]	Prospective, 6 months follow up	141	Early PSCI	MoCA	SBP	↑Acute phase SBP →↓ Cognitive performance
He, M. et al. [187]	Prospective, 12 months follow up	796		MoCA	SBP, DBP Ultra-short BPV Daily BPV Long-term BPV	High and lower SBP → Risk of early PSCI↑
Levine, D.A. et al. [188]	Cross section, 90 days after stroke	432	Non-demented No cognitive impairment	Modified MMSE, Trails A, and Trails B	SBP, DBP, PP, MAP	Lower DBP →Lower trails B score
Yamamoto, Y. et al. [189]	Prospective, 4.1 years follow up	249		MMSE	Home BP (HBP)	↑HBP → Risk of late PSCI↑
Sachdev, P.S., et al. [190]	Cross section, 3-6 months after stroke Case control	169 stroke 103 Control		Comprehensive Neuropsychological Assessment	Diagnosis of Hypertension	Hypertension is not a risk factor for PSCI
Ihle-Hansen, H. et al. [191]	Prospective, 1 ear follow up	166	First-ever stroke	Diagnosis of MCI Diagnosis of dementia	SBP, DBP	No association of BP level and dementia or MCI
Lu, Z.H. et al., [192]	Prospective, 6 months follow up	232	First-ever stroke	MoCA	Diagnosis of hypertension	Hypertension with hyperhomocysteinemia → Risk of early PSCI↑; but not in HTN only patients
Geng, S., et al. [193]	Prospective, 12 months follow up	796		MoCA	SBP, DBP SBPV, DBPV	↑early SBPV→ Risk of late PSCI↑
Lee, J.H. et al. [194]	Prospective 3 months follow up	36	Lacunar infarction	MMSE, Controlled Oral Word Association Test, Digit Symbol Coding test	DBPV, SBPV	↑early SBPV→ Risk of early PSCI↑, especially frontal lobe dysfunction
Kim, Y., et al. [195]	Prospective, 2.6 years follow up	746		MMSE, MoCA	SBP, DBP SBPV, DBPV	↑BPV→ Risk of late PSCI ↑

↑, increase; ↓. decrease; → leads to.

## Data Availability

The data was available upon reasonable email request.
